# Protein kinase CK2: a potential therapeutic target for diverse human diseases

**DOI:** 10.1038/s41392-021-00567-7

**Published:** 2021-05-17

**Authors:** Christian Borgo, Claudio D’Amore, Stefania Sarno, Mauro Salvi, Maria Ruzzene

**Affiliations:** 1 Department of Biomedical Sciences, University of Padua, Padua, Italy; 2 CNR Institute of Neuroscience, University of Padua, Padua, Italy

**Keywords:** Medicinal chemistry, Target identification, Drug development

## Abstract

CK2 is a constitutively active Ser/Thr protein kinase, which phosphorylates hundreds of substrates, controls several signaling pathways, and is implicated in a plethora of human diseases. Its best documented role is in cancer, where it regulates practically all malignant hallmarks. Other well-known functions of CK2 are in human infections; in particular, several viruses exploit host cell CK2 for their life cycle. Very recently, also SARS-CoV-2, the virus responsible for the COVID-19 pandemic, has been found to enhance CK2 activity and to induce the phosphorylation of several CK2 substrates (either viral and host proteins). CK2 is also considered an emerging target for neurological diseases, inflammation and autoimmune disorders, diverse ophthalmic pathologies, diabetes, and obesity. In addition, CK2 activity has been associated with cardiovascular diseases, as cardiac ischemia–reperfusion injury, atherosclerosis, and cardiac hypertrophy. The hypothesis of considering CK2 inhibition for cystic fibrosis therapies has been also entertained for many years. Moreover, psychiatric disorders and syndromes due to CK2 mutations have been recently identified. On these bases, CK2 is emerging as an increasingly attractive target in various fields of human medicine, with the advantage that several very specific and effective inhibitors are already available. Here, we review the literature on CK2 implication in different human pathologies and evaluate its potential as a pharmacological target in the light of the most recent findings.

## Introduction

### CK2 general features

CK2 (previously called casein kinase 2 or CK-II) is one of the first identified protein kinases.^1^ It phosphorylates hundreds of physiological substrates,^2^ and is one of the major contributors to the generation of the human phospho-proteome.^3^

Structurally, mammalian CK2 is a tetrameric enzyme, composed of two catalytic and two regulatory subunits. The catalytic ones might be represented either by α or α′, very similar but encoded by two different genes, *CSNK2A1* and *CSNK2A2*, respectively, while only one human CK2 regulatory subunit exists, β, encoded by the *CSNK2B* gene. The regulatory functions of β are limited to preserving the enzyme stability and driving the selection of substrates.^4^ In fact, CK2 is constitutively active, and catalytically competent also in its monomeric form.^4^

### CK2 functions and involvement in signal transduction

**Fig. 1:**
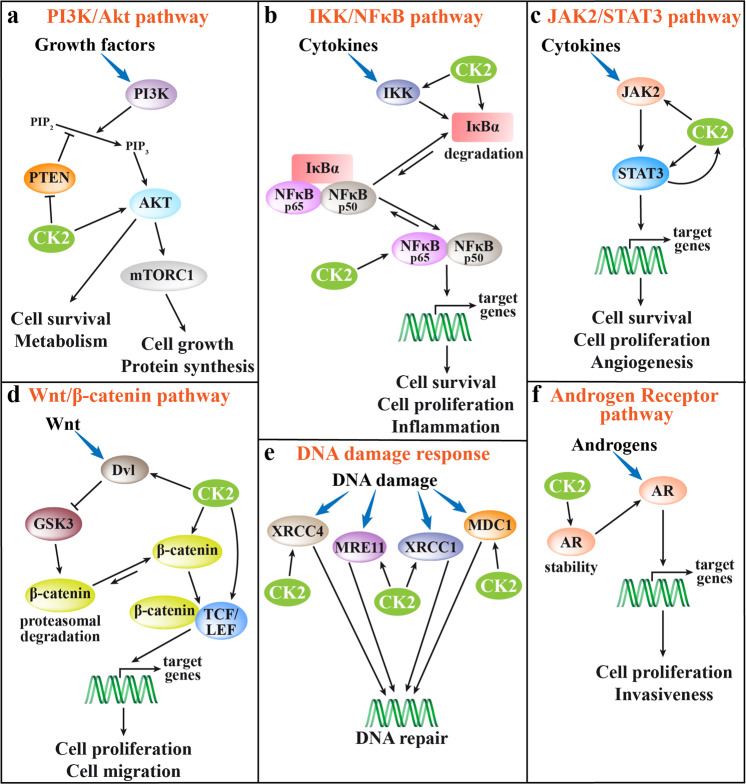
Depiction of the most relevant CK2 interventions on cellular signaling pathways. Double arrows indicate a dynamic equilibrium that moves toward the longest arrow direction; inhibitions are indicated by bar-headed arrows. **a** PI3K/Akt pathway: CK2 is known to directly potentiate Akt functions, but it also inhibits PTEN, thus preventing its downregulating functions. **b** IKK/NFκB pathway: CK2 induces IκBα degradation thus reducing its inhibitory action, and stimulates IKK and the p65 subunit of NFkB. **c** JAK2/STAT3 pathway: CK2 directly activates JAK2 and STAT3 and, in turn, CK2 expression is under the control of STAT3. **d** Wnt/β-catenin pathway: CK2 activates Dvl, thus inhibiting the GSK3-mediated degradation of β-catenin, and phosphorylates β-catenin, promoting its stability; moreover, its phosphorylation of TCF/LEF stimulates the β-catenin/LEF complex formation and transcriptional activity. **e** DNA damage response: CK2 phosphorylates the indicated proteins to improve their DNA repair activity. **f** Androgen receptor (AR) pathway: CK2 activity increases AR protein stability, leading to promote the AR-dependent transcriptional activity

In signal transduction, CK2 is defined as a “lateral player”.^5,6^ In fact, being constitutively active, it does not respond to a figuratively “vertical” stimulus coming from outside the cell. It is instead already present and ready to play its “horizontal” function on pathways that are otherwise activated. Among the many CK2 substrates, components of diverse signaling pathways are present, implying that CK2 controls important cellular processes, frequently producing abnormal responses and contributing to pathological phenotypes. Its intervention that might cause dysregulation relevant for human diseases has been dissected in several signaling pathways. The ones with the most well-defined role of CK2 are shown in Fig. 1. Figure 1a schematically describes the multilevel intervention of CK2 on the PI3K (phosphoinositide 3-kinase)/Akt pathway:^7,8^ CK2 directly phosphorylates Akt1 at Ser129, thus promoting its activity and stabilizing the phosphorylation of the PDK1 (phosphoinositide-dependent kinase 1)-dependent activation site Thr308. Moreover, CK2 phosphorylates PTEN, with the effect of inhibiting its phosphatase activity and preventing the downregulation of PI3K-dependent signaling. The participation of CK2 in the IKK (IκB kinase)/NFκB pathway^9^ (Fig. 1b) is based on several targets, including IκBα (inhibitor of NFκB), whose phosphorylation is increased by CK2 both directly and through the activation of IKK. The phosphorylation of IκBα promotes its degradation, and the consequent NFκB release from the inhibitory complex with its final nuclear translocation. In addition, Ser529 of the NFκB p65 subunit is also phosphorylated by CK2, with the effect of increasing NFκB p65 transcriptional activity.^10^ On JAK2/STAT3 pathway (Fig. 1c), CK2 targets both STAT3^11^ and JAK2,^12^ resulting in a final amplification of cytokine signals. Interestingly, CK2 itself has been found under the control of STAT3.^13^ The Wnt/β-catenin pathway (Fig. 1d) is another signaling with a multisite regulation by CK2,^9^ which intervenes at the level of dishevelled (Dvl; thus reducing its GSK3-mediated degradation of β-catenin), β-catenin (to promote its nuclear translocation and transcriptional activity), and the transcription factor TCF/LEF. CK2 is known to increase the DNA repair in response to damage signals^14^ (Fig. 1e); the mechanism implies the phosphorylation of several proteins, such as XRCC4 (crucial for the nonhomologous end-joining, NHEJ, the major DNA double-strand break repair pathway), and XRCC1 (promoting DNA single-strand break repair); in general, the effect of the CK2-dependent phosphorylation is an increased association to DNA–repair protein complexes. On the signaling elicited by the androgen receptor (AR) stimulation (Fig. 1f), CK2 activity has been shown essential for the stability of the receptor protein, and therefore to support the AR transcriptional action.^15,16^ Some roles of CK2 have been reported also in other signaling pathways, such as Hedgehog,^17^ TNF-α,^18^ Notch1,^19^ and Tyr-kinase receptors.^18,20^ The degree by which CK2 potentiates each signal depends of course on its expression/activity level, becoming prominent in cancer cells, where CK2 is usually overexpressed^3,6^ (see below).

### CK2 inhibitors

Given its implication in several dysregulated pathways in human pathologies, CK2 is considered as a drug target. An advantage is represented by the wide spectrum of inhibitors already available and potentially in the hands of clinicians. Table 1 lists the different classes of CK2 inhibitors on the basis of their mechanisms of action; the most representative compounds for each class/subclass, and the corresponding references, are also indicated. Despite the majority of them being ATP competitive, they are often very specific, due to the distinct features of the CK2 catalytic site, which is smaller than in most other protein kinases (where it is instead too large for making stable interactions with the inhibitors).^21^ The specificity is further increased in the case of the so-called bi-substrate inhibitors (as ARCs^22^ or K137-E4^23^), which are competitive for both the phosphate donor nucleotide and the phosphate acceptor peptide. Other compounds are allosteric inhibitors, acting at the interface between α and β subunits (as Pc^24^), while in some cases the mechanism of action is poorly understood (as POM^25^), or is based on specific CK2 targets (as CIGB-300^26^).

**Table 1:** Classification of CK2 inhibitors

Class of the inhibitor	Subclass	Representative compounds	References
ATP-competitive inhibitors	Polyhalogenated benzimidazoles and benzotriazoles	TBB	^307^
		TDB	^308^
		DMAT	^309^
	Flavonoids	Quercetin	^310^
	Coumarins	Ellagic acid	^311^
	Anthraquinones	Quinalizarin	^312^
	Pyrazolotriazines	Compound 9e	^28^
	SRPIN803 derivatives	Compound 4	^313^
	Carboxyl acid derivatives	TBCA	^314^
		IQA	^310^
		**CX-4945**	^315^
		CX-5011	^27^
	Triazol bromoguaiacol derivatives	GO289	^29^
Peptide-competitive inhibitors		**CIGB-300**	^26^
Allosteric inhibitors	α/β interface targeting	Pc	^24^
		POMs	^25^
Bi-substrate inhibitors		ARCs	^22^
		K137-E4	^23^
Multi-target inhibitors	CK2 and HDAC targeting		^316^
	CX-4945 + CisPt		^317^

Compounds in human clinical trials are in bold

*TBB* 4,5,6,7-tetrabromo-2-azabenzimidazole, *TDB* 1-(β-D-2′-deoxyribofuranosyl)-4,5,6,7-tetrabromo-1H-benzimidazole, *DMAT* 2-dimethylamino-4,5,6,7-tetrabromo-1H-benzimidazole, *TBCA* tetrabromo-cinnamic acid, *IQA* [5-oxo-5,6-dihydro-indolo(1,2-a)quinazolin-7-yl]acetic acid, *POMs* polyoxometalates, *HDAC* histone deacetylase, *CisPt* cisplatin

Many inhibitors are very effective, with Ki in the nanomolar or sub-nanomolar range, as for example CX-4945 (Ki 0.17 nM^27^), CX-5011 (Ki 0.22 nM^27^), compound 9e (Ki 0.35 nM^28^), and GO289 (Ki 7 nM^29^). This latter has also the advantage to be more effective in cells, compared to CX-4945,^30^ and to avoid methuosis induction, which is instead observed in response to CX-4945 (refs. ^31,32^) and CX-5011.^32^ More comprehensive descriptions, with detailed inhibition parameters, can be found in recent reviews on CK2 inhibitors.^33–35^

Two CK2 inhibitors, CX-4945 and CIGB-300, are already in human trials as anticancer drugs. CX-4945 (commercial name Silmitasertib) has been designated as an orphan drug by FDA for the treatment of cholangiocarcinoma, and several clinical studies (phases I/II) are ongoing with it (https://clinicaltrials.gov/ct2/results?cond=&term=CX-4945&cntry=&state=&city=&dist=). CIGB-300, which is a different type of inhibitor, preventing the CK2-dependent phosphorylation of specific substrates, is under investigation for cervical cancers (https://clinicaltrials.gov/ct2/results?cond=&term=cigb-300&cntry=&state=&city=&dist=).^26^

Recent reviews^36–38^ summarize several studies confirming the efficacy of CK2 inhibitors in different cellular and animal models of diseases. The major findings with the two CK2 inhibitors already in clinical trials (CX-4945 and CIGB-300) are summarized in Table 2.

**Table 2:** Major findings reported for the use of CX-4945 and CIGB-300 in cell and in vivo

	CIGB-300	CX-4945
In vitro studies	Antiproliferative effect and apoptosis induction in different cancer cell lines^318–321^ Angiogenesis inhibition^323^ Adhesion, migration and invasion reduction in different cancer cell lines^56,321^	Antiproliferative effect and apoptosis induction in different cancer cell lines^43,98,100,319,324–330^ Angiogenesis inhibition^328^ Adhesion, migration and invasion reduction in different cancer cell lines^77,333,334^ Methuosis induction^31,32,305^ Inhibition of cell differentiation^267,268,335,336^ Overcoming cancer cells chemoresistance^37,42^ Suppression of DNA damage repair mechanism^337^ Intracellular Ca^++^ dynamics regulation^338^ Splicing regulation^339^ Reduction of pro-inflammatory cytokines release^106,163^ Stimulation of insulin release^283^ Regulation of anion channels activity^230,231^ Potential anti-COVID-19 treatment^199^
In vivo studies	Anti-tumor effect in murine mouse models^56,318,319^ Angiogenesis inhibition^56,323^	Anti-tumor effect in murine models^96,324,327–332^
Human studies	Tumor reduction in women affected by cervical cancer^322^ Cancer clinical trials^a^: NCT01639625, NCT01639638	Cancer clinical trials^b^: NCT01199718, NCT00891280, NCT03897036, NCT02128282, NCT03904862, NCT03571438 COVID-19 clinical trials^b^: NCT04668209, NCT04663737

^a^https://clinicaltrials.gov/ct2/results?cond=&term=cigb-300&cntry=&state=&city=&dist=.

^b^https://clinicaltrials.gov/ct2/results?cond=&term=CX-4945&cntry=&state=&city=&dist=.

## CK2 in human diseases

### CK2 in cancer

Cancer is definitely the human disease where a role for CK2 has been more widely and longer documented. The first connection of CK2 to cancer dates back to 1995 when Seldin and Leder discovered that a lymphoproliferative syndrome was associated with CK2 overexpression, and that the co-expression of CK2 catalytic subunit and c-myc was capable of transforming lymphocytes.^39^ Since then, CK2 was found overexpressed in several cancer cells in comparison to healthy counterparts, and a plethora of studies was published supporting functions of CK2 in the pathogenesis of cancer.

The identified mechanisms rely on the CK2 capability of potentiating other oncogenic signaling (see above, concept of “lateral player”). In a recent large-scale affinity chromatography-mass spectrometry (MS) study depicting a comprehensive interaction map for the human kinome, CK2 has been identified as one of the main kinases establishing direct interactions with cancer-associated proteins.^40^

**Fig. 2:**
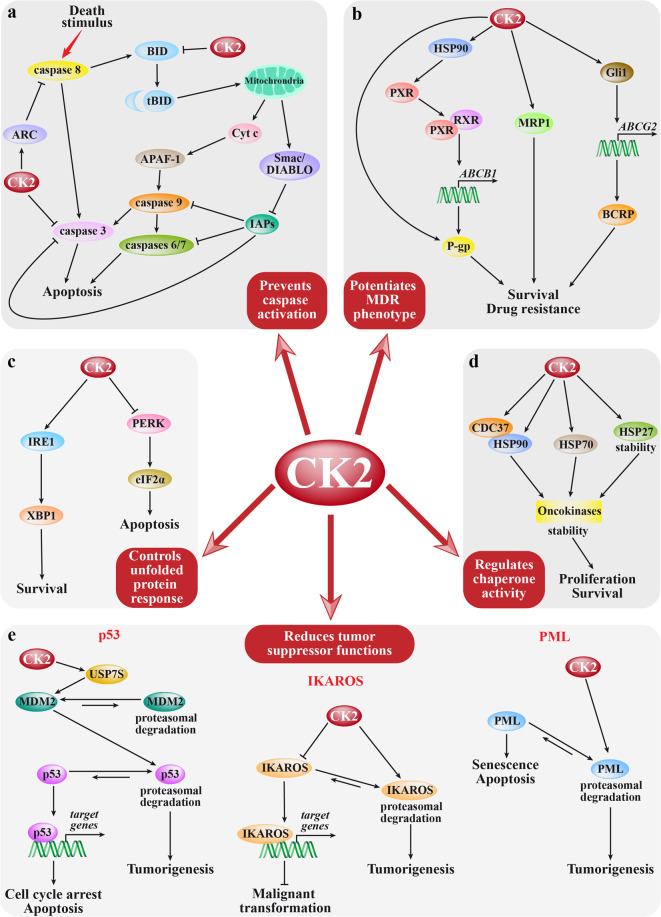
CK2 roles in cancer. Signaling pathways by which CK2 exerts its specific functions in cancer cells are depicted. For each pathway, CK2 targets are shown only in case their effects in tumorigenesis have been dissected (not showing CK2 substrates whose phosphorylation does not produce a well-defined effect). Double arrows indicate a dynamic equilibrium that moves toward the longest arrow direction; inhibitions are indicated by bar-headed arrows. **a** Major mechanisms by which CK2 prevents caspase activation. CK2 phosphorylation of BID prevents its cleavage to the truncated form (tBID) and its consequent migration to the mitochondria; this event blocks the apoptotic cascade dependent on the cytosolic release of proapoptotic factors cytochrome c (Cyt c), an activator of caspase 9 via APAF-1 (apoptotic protease activating factor-1), and Smac/DIABLO, a repressor of IAP (inhibitor of apoptosis) proteins. Furthermore, CK2 directly phosphorylates and prevents the activation of caspase-3, and promotes the action of the caspase inhibitor ARC, which blocks caspase 8. **b** CK2 effects on multidrug resistance (MDR). CK2 reduces the cancer cell response to chemotherapeutic drugs by promoting the expression of the three major drug extrusion pumps, namely MRP1, P-gp, and BCRP. MRP1 and P-gp are also directly activated by CK2. **c** Major actions of CK2 on the unfolded protein response pathway. CK2 acts on different branches of the unfolded protein response, with the effect of preventing the final apoptotic outcome (by blocking the PERK signaling) and driving towards the survival response (by supporting the IRE1 signaling). **d** CK2 regulation of chaperone proteins. CK2 directly controls the activity of HSP70 and CDC37 (HSP90 co-chaperone), and protects HSP27 from degradation. These chaperones, in turn, stabilize and maintain the activity of oncogenic proteins, especially protein kinases (oncokinases). **e** Major mechanisms of CK2 control on tumor suppressor proteins. CK2 promotes p53 degradation through the phosphorylation of the ubiquitin-specific peptidase 7 (USP7S); this in turn stabilizes MDM2 with the final effect of targeting p53 to the proteasome. IKAROS is directly phosphorylated by CK2, reducing its DNA-binding affinity and promoting its degradation. CK2 also directly phosphorylates PML; this drives its proteasome-mediated degradation, and finally reduces its function of promoting senescence and apoptosis

Figure 2 summarizes the main mechanisms by which CK2 sustains tumorigenesis, and the involved pathways. CK2 has a prominent antiapoptotic role, mainly played by counteracting the caspase action^41^ (Fig. 2a). It phosphorylates caspase substrates on residues in the proximity of the cleavage sites, thus preventing the cleavage and the generation of the proapoptotic truncated forms; Fig. 2a shows one of the most important examples, BID, which, if phosphorylated, is not cleaved to tBID and cannot trigger the release of proapoptotic factors from the mitochondria. CK2 interferes with the caspase action also directly (as on caspase-3) or by promoting the action of caspase inhibitors (as the apoptosis repressor with caspase recruitment domain, ARC). CK2 potentiates the multidrug resistance (MDR) phenotype^42^ (Fig. 2b), by enhancing the expression and/or the activity of the extrusion pumps P-gp, MRP1, and BCRP, thus favoring drug efflux from tumor cells. Moreover, CK2 supports other processes responsible for cancer drug resistance, such as several mechanisms of DNA repair^14,18^ (see Fig. 1e). CK2 protects from unfolded protein response (Fig. 2c),^43,44^ acting both on IRE1 (activating its XBP1-mediated pro-survival function) and PERK (inhibiting its eIF2α-mediated promotion of apoptosis). Of special importance, CK2 controls the activity of chaperone proteins (Fig. 2d), especially those committed to the oncokinases, such as the HSP90 co-chaperone CDC37,^45^ but also HSP70^46^ and HSP27^47^; by this function, CK2 magnifies the spectrum of its targets and controlled proteins far beyond its direct substrates. CK2 contributes to malignancies also by reducing the amount and/or the activity of tumor suppressor proteins (Fig. 2e). On the action of CK2 on p53 tumor suppressor functions, several findings have been reported. The effects of the direct CK2-dependent phosphorylation of p53 or of its negative regulator MDM2 are still elusive.^48,49^ However, other mechanisms of CK2 regulation of the MDM2/p53 axis have been proposed (Fig. 2e): CK2 phosphorylates a specific isoform of the ubiquitin-specific peptidase 7, thus stabilizing MDM2, with the final effect of p53 downregulation.^50^ CK2 also phosphorylates IKAROS at multiple sites, promoting its proteasomal degradation and reducing its binding to DNA, with the final effect of an impaired transcription of target genes^51^ (Fig. 2e). CK2 phosphorylates PML (Fig. 2e), driving it to ubiquitin-mediated degradation^52^; by this mechanism CK2 fosters senescence escape of tumor cells.^13^ Finally, also the PTEN tumor suppressor is under the control of CK2 (see Fig. 1a), which inhibits its activity and repressive function on the PI3K-dependent growth signals.^7,8^

Besides the pathways illustrated in Fig. 2, which specifically refer to functions in cancer, the CK2 tumorigenic action is also played on the survival and proliferation pathways depicted in Fig. 1, which become aberrantly potentiated by CK2 in cancer, due to its overexpression.^18,41,53–55^ By all these mechanisms, CK2 supports the cancer hallmarks and the tumorigenic phenotype, promoting migration and invasion,^36,56–60^ EMT (epidermal–mesenchymal transition),^61^ aggressiveness,^62,63^ angiogenesis,^64^ and adaptation to hypoxia.^65^ A role in the metabolic rewiring of tumor cells is also emerging.^57,66–70^

The CK2 isoform which majorly contributes to the oncogenic phenotype is still a matter of debate. The observation that an unbalanced expression of α or α′, not compensated by β, may promote transformation was initially reported in Ha-ras-transfected fibroblasts.^71^ The view of the monomeric CK2 as more related to the tumor phenotype than tetrameric CK2 was later supported by other observations: β has functions in cell plasticity^61^ and its decrease correlates with EMT promotion and increased metastatic risk in breast cancer^72^ and clear cell renal cell carcinoma.^73^ Consistent with a “tumor suppressor-like” function of β, the phosphorylation of CK2 substrates that are crucial for its antiapoptotic role in cancer, like caspase-3, is prevented by β.^74^ Also, the role of CK2 in drug resistance has been found related to the overexpression of α but not β, at least in some MDR cell clones.^75,76^ However, also pro-tumorigenic functions have been reported for β: in the immortalized neurons GN11, β promotes migration.^77^ Interestingly, there are also observations supporting different contribution to the tumor phenotype of the two catalytic isoforms: α′ was found selectively important for osteosarcoma cell proliferation and survival,^78^ and the proliferation rate of tumor cells in a murine model of glioblastoma.^79^ In contrast, the copy number of the genes for α (*CSNK2A1*) and β (*CSNK2B*) were found to display gain in breast tumors (~30% and 20%, respectively),^80^ while much fewer gains were found on gene for α′ (*CSNK2A2*).^80^ Intriguingly, in the already mentioned study on GN11 cells, α′, while promoting adhesion, reduces migration. It is conceivable that the pro-tumor role of each specific isoform cannot be objectively described, being dependent on the type of cancer, and possibly on selected targets present in those tumor cells and specifically requiring a CK2 isoform for phosphorylation.

As far as the cancer types where CK2 was found relevant, hematological malignancies are probably the most studied. Overexpression and specific pro-oncogenic functions of CK2 have been reported in leukemias, such as T-ALL,^81^ B-ALL,^82,83^ AML,^84,85^ CLL,^86^ CML,^87^ in lymphomas,^11,88^ and multiple myeloma.^44,89^ Several reviews summarize the findings, with the specific targets and roles proposed for CK2 in the different blood cancers.^51,53,90–93^

Among solid tumors, overexpression and/or important roles have been found for CK2 in glioblastoma,^94–98^ medulloblastoma,^99,100^ prostate cancers,^101–103^ ovarian cancers,^104–107^ breast cancers,^72,108–112^ head and neck squamous cell cancers,^62,113,114^ lung cancers,^113,115–117^ melanoma,^118,119^ renal cell carcinoma,^73,120–122^ bladder cancers,^123,124^ pancreatic cancers,^125,126^ cholangiocarcinoma,^36,127^ esophageal cancers,^128,129^ gastric cancers,^130–133^ hepatocellular carcinoma,^134–137^ mesothelioma,^138^ cervical cancers,^139–141^ and other squamous cell carcinoma.^142^ In this list, the references quote the first chronological evidence and the most relevant findings of CK2 overexpression or fundamental roles in the indicated type of tumor (not considering the many publications dealing with CK2 inhibitor treatment, unless conclusions highlighted specific CK2 functions in the treated tumor).

The mechanisms of the strong CK2 involvement in tumors have been highlighted in excellent reviews.^54,143–146^ Moreover, two analyses were published extracting data on the CK2 transcript expression from the Oncomine Database.^125,147^ Interestingly, the CK2 transcripts analyses disclosed increased levels of CK2 subunits in most cases, and in general worse prognosis was associated with overexpression. But, unexpectedly, also downregulation was observed in some cases, as in testis cancer. However, these studies analyzed CK2 only at the mRNA levels, and they do not provide any evidence of the actual CK2 subunit protein levels in cancer specimens; thus, these conclusions should be not directly expanded to protein/activity.

No gain of function mutation is known, so far, accounting for the oncogenic properties of CK2, and the upregulation of CK2 activity in cancer cells is mainly due to an increased protein amount. The reason for the overexpression is still unknown. Gene amplification has been reported for one-third of glioblastoma cases^98^ and in breast tumors (30% *CSNK2A1*, α gene, and 20% *CSNK2B*, β gene);^80^ however, activity does not seem correlated with the copy number.^95^ For a long time CK2 amount was considered mainly regulated at the protein level,^148^ but emerging evidence of different mRNA amounts suggests the possibility of transcriptional regulation or increased transcript stability.^125,147^

CK2 cannot be properly defined as an oncogene, since it is ubiquitously expressed and not specifically linked to oncological conditions. However, cancer cells are addicted to CK2 in a sort of “non-oncogene addiction”,^149^ since they rely on CK2 for their survival much more than normal cells.^6^ This observation is the basis of the numerous studies on the use of CK2 inhibitors in animal models and clinical trials in humans.

The mechanism underlying the higher CK2 levels observed in tumors can be only a matter of speculations: the event appears to be mainly based on a stochastic mechanism driven by tumor-promoting selection of those cells which by chance express higher CK2 and are therefore favored over the others in surviving, proliferating, and propagating their features.^3^

### CK2 in neurodegenerative diseases

**Fig. 3:**
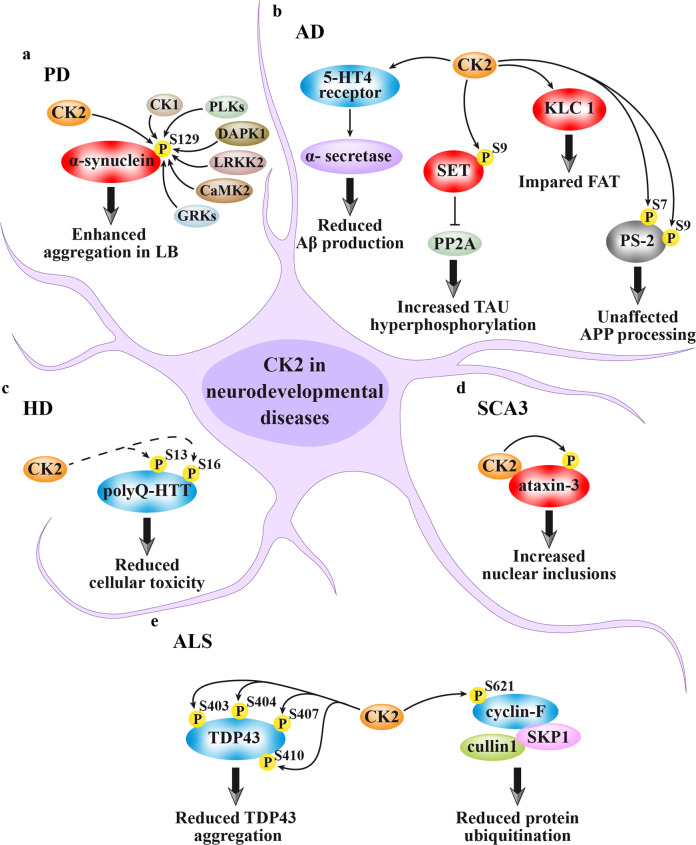
CK2 targets in neurodegenerative diseases. Red shapes denote targets that mediate CK2 pathological functions, blue shapes are proteins by which CK2 may exert a protective function against the disease, gray shapes indicate that the CK2-dependent phosphorylation has no disease-related effect. **a** Parkinson’s disease (PD): CK2 promotes α-synuclein aggregation in Lewis bodies (LB) by phosphorylating its Ser129, but this site is also target of other protein kinases. **b** Alzheimer’s disease (AD): CK2 is responsible for the 5-HT_4_ receptor-stimulated induction of α-secretase activity, which in turn reduces the Aβ (amyloid β-peptide) production, through the non-amyloidogenic pathway of amyloid precursor protein (APP) processing. However, CK2 induces tau hyperphosphorylation, through the phosphorylation of SET, an inhibitor of the PP2A phosphatase, and its consequent cytosolic localization and binding to PP2A. Moreover, CK2 phosphorylates KLC 1, causing FAT impairment. Another AD-related CK2 target is PS-2, whose phosphorylation, however, does not affect the APP processing. **c** Huntington’s disease (HD): the HTT sites Ser13 and Ser16, found hypo-phosphorylated in the polyQ-HTT mutant, are increased by CK2 through a direct or indirect mechanism (dashed arrows), and this reduces cellular toxicity. **d** Spinocerebellar ataxia type 3 (SCA3): CK2 associates to and phosphorylates ataxin-3, thus promoting its nuclear localization and stabilization, and enhancing the formation of inclusions. **e** Amyotrophic lateral sclerosis (ALS): CK2 is a potential kinase of TDP43, the major component of protein aggregates in motor neurons, whose phosphorylation decreases its propensity to aggregate. Moreover, CK2 phosphorylates cyclin F, thus negatively controlling the E3 ligase activity of the SKP1/cullin1/F‐box (SCF)‐E3 ligase complex, and finally reducing the aberrant proteins ubiquitination typically observed in ALS

The several neurodegeneration-related CK2 targets identified so far are schematically shown in Fig. 3. The effects of CK2 on these targets may be either pathogenic (red shapes in Fig. 3) or protective against the disease (blue shapes). The specific CK2 actions for each neurodegenerative disease are described here below.

#### Parkinson’s disease (PD)

The hypothesis of a CK2 implication in the pathogenesis of PD refers to its role in phosphorylating α-synuclein at the Ser129 (Fig. 3a). This is an intrinsically disordered protein, whose aggregates are the main component of the Lewis bodies (LB), typically present in the *substantia nigra* of PD patients.^150^ Of note, mutations in α-synuclein gene *SNCA*, as duplications, triplications, and point mutations, are responsible for the autosomal dominant form of PD. α-synuclein phosphorylation has been early hypothesized as having a role in the protein toxicity and propensity to aggregate, since it was found phosphorylated at nearly 90% at the Ser129 site in LB (whereas this residue was only phosphorylated at 4% or less in normal brain).^151^ The identification of the kinase responsible for this phospho-site was therefore considered crucial because it could represent a pharmacological target to treat the disease. CK2 has been one of the first enzymes to be hypothesized as α-synuclein Ser129 kinase.^152^ Indeed, CK2 colocalizes with α-synuclein in the LB, phosphorylates its Ser129 in vitro and in cells, and the phospho-site is sensitive to CK2 inhibitors.^152^ So, in 2007 CK2 was suggested to be the main α-synuclein Ser129 kinase in the brain.^153^ However, 2 years later, by different in vitro and in vivo experimental approaches, a newly described family of acidophilic kinases, the Polo-like kinases (PLKs), were identified as the main family of kinases responsible for this phosphorylation, with primary function for the PLK2 isoform and, to a lesser extent, for PLK3.^154,155^ The higher phosphorylation efficiency of PLKs compared to CK2 has been also reproduced in vitro using recombinant proteins, and is attributable to the fact that the target sequence (YEMP**S**EEG) fits better for PLKs (ExxS/TxE/D) than for CK2 consensus sequence (SxxE/D).^156–158^ Indeed, two widely used CK2 inhibitors, TBB and CX-4945, also affect PLK2/PLK3 activity, thus explaining the previous misleading conclusion on the main role of CK2 in α-synuclein phosphorylation.^159^

However, the story has not ended and some findings suggest it is more complex than it seems. In fact, PLK2 knockout rats show a not over 50% decrease in Ser129 phosphorylation, and the residual phosphorylation is not sensitive to a specific PLK1-3 inhibitor, suggesting that this site is targeted by multiple kinases in vivo.^160^ To date, a large number of protein kinases have been found involved, including CK2, CK1, PLK2, PLK3, G-protein-coupled receptor kinases, Ca^2+^/calmodulin-dependent kinase 2, leucine-rich repeat kinase 2, and death-associated protein kinase 1, and, interestingly, some evidence suggests that the biological effects of Ser129 phosphorylation might be different, dependent on the specific phosphorylating kinase.^151^ However, it is also worth mentioning that controversial results have been published on the role of α-synuclein phosphorylation in aggregation and toxicity, thus raising perplexity on the opportunity of its therapeutic inhibition.^151^

#### Alzheimer’s disease (AD)

AD is a progressive neurodegeneration, characterized by the presence in amyloid plaques of amyloid β-peptide (Aβ), which is produced by sequential cleavage of amyloid precursor protein (APP) by the β- and the γ-secretases.^161^

There are numerous evidence for CK2 implication in AD. An increase of CK2 activity has been reported in AD.^162,163^ This has been explained, at least in part, by a direct allosteric activation of CK2 by the Aβ peptide, as shown in vitro;^164^ however, its relevance in AD pathology is far from being fully understood.

CK2 is responsible, at least in vitro, for the direct phosphorylation of Ser7 and Ser9 of Presenilin-2 (PS-2), a protein that is part of the γ-secretase complex and involved in APP processing^165,166^ (Fig. 3b). However, a mutational approach suggests that the phosphorylation of these sites would not affect APP processing.^166^

CK2 is instead necessary, through the 5-hydroxytryptamine 4 (5-HT_4_) receptor, for the α-secretase activity that produces the soluble APPα^167,168^ (Fig. 3b). Since an increased α-secretase activity would reduce Aβ peptide production and amyloid plaque formation, an enhancement of CK2 activity would be desirable to treat AD. However, other lines of evidence suggest, on the opposite, that CK2 inhibition would be beneficial in AD. One of the mechanisms by which CK2 would be part of the pathogenic process is based on its ability to phosphorylate Ser9 of SET, an inhibitor of phosphatase PP2A^162^ (Fig. 3b). SET, when phosphorylated by CK2, moves to cytoplasm where it binds and inhibits PP2A, leading to tau hyperphosphorylation; this has been found accompanied by cognitive impairments in mouse models of CK2 overexpression in hippocampus.^162^

Moreover, CK2 activity was found relevant for the impairment of the fast axonal transport (FAT, the bidirectional movement of membranous organelles along microtubules driven by molecular motors), which is defective in AD^169^ (Fig. 3b). It was found that treatment of isolated axoplasm with the amyloid peptide Aβ42 induces CK2 activation; this promotes increased phosphorylation of kinesin-1 light chains (KLC 1), which leads to the release of the vesicle cargo from motor proteins, and axonal transport inhibition.^170^ Notably, CK2 pharmacological inhibition prevents the effect of Aβ42 on FAT, and the inhibitory effect of Aβ42 on FAT can be recapitulated by the perfusion of the axon with recombinant CK2.^170^

In summary, CK2 is undoubtedly involved in AD, but its functions, according to the different reports, range from irrelevant^166^ to instrumental^162–164,169,170^ or even unfavorable^167,168^ for the pathogenesis of the disease, and the evidence for therapeutic employment of CK2 inhibitors is still very elusive.

#### Neurodegenerative diseases caused by polyglutamine expansion

There are several neurodegenerative disorders caused by expanded polyglutamine repeats. Huntington’s disease (HD) is a dominant neurodegenerative disease caused by an expanded CAG repeat in the *HTT* gene encoding the Huntingtin protein (HTT). The mutant variant, with an expanded polyglutamine (polyQ) tract, is toxic and responsible for the disease.^171^ The first evidence of a CK2 implication in HD was obtained by screening the expression of a subset of signaling molecules in HEK293 cells overexpressing the NMDAR receptor along with wild type (w.t.; 15Q) HTT or polyQ‐HTT. CK2 was found upregulated in cells expressing polyQ‐HTT, and this was considered as a cellular response to counteract polyQ‐HTT toxicity; consistently, CK2 inhibition enhanced polyQ‐HTT toxicity.^172^ A protective role of CK2 in HD was further confirmed showing that HTT phosphorylation at two N-terminus sites, Ser13 and Ser16, affects the protein conformation and subcellular localization^173^ (Fig. 3c). PolyQ‐HTT is hypo-phosphorylated at these sites compared to w.t. HTT, and displays a more cytoplasmic localization, while phospho-mimetic mutant restores the nuclear localization and reduces the cellular toxicity of polyQ‐HTT.^173^ Although CK2 inhibitors reduce Ser13 and Ser16 phosphorylation, further work will be necessary to verify if HTT is a direct CK2 substrate or an indirect target. The latter hypothesis is the most likely, as the N-terminal sequence of HTT lacks the CK2 consensus acidic residues (KAFE**S**_**13**_LK**S**_**16**_FQQQ). However, a recent high-throughput screening to find out molecules able to enhance Ser13 and Ser16 phosphorylation identified the molecule N6-furfuryladenine, that was able to restore HTT phosphorylation and exhibited a protective role.^174^ The effect of N6-furfuryladenine has been linked to the metabolic production of KTP, an ATP analog that can be used by CK2 for the enzymatic phosphorylation. Consistently, N6-furfuryladenine is ineffective in the presence of CK2 inhibitors. In summary, all the current evidence supports a protective role for CK2 in HD disease.

However, this cannot be extended to other polyglutamine expansion diseases. Spinocerebellar ataxia type 3 (SCA3) is an autosomal dominant neurodegenerative disease caused by expansion of a glutamine-encoding CAG repeat in the *ATXN3* gene. The product of this gene, ataxin-3, associates to and is phosphorylated by CK2^175^ (Fig. 3d), which induces its nuclear localization and stabilization, and enhances the formation of inclusions.^176,177^ Accordingly, CK2 inhibition leads to an almost complete disappearance of nuclear inclusions.^176^ In this view, CK2 would represent a potential pharmacological target to treat SCA3 disease.

#### Amyotrophic lateral sclerosis (ALS)

ALS is a progressive neurodegenerative disorder characterized by the death of upper and lower motor neurons leading to the loss of voluntary muscle function. The disease is typically characterized by the presence of protein aggregates in the cytoplasm of motor neurons; the TAR DNA-binding protein (TDP43) is the main component of these pathological deposits.^178^ A wide range of posttranslational modifications (PTMs), including phosphorylation, ubiquitination, acetylation, sumoylation, and others, have been suggested to regulate the propensity of this protein to form aggregates.^179^ CK2 is one of the kinases potentially involved in the phosphorylation of TDP43, in particular at Ser403, Ser404, Ser409, and Ser410 (Fig. 3e). Increased phosphorylation of these sites, observed in response to CK2α overexpression in cultured cells, is associated with a decrease in aggregation propensity of truncated forms of TDP43, an effect prevented by the addition of a CK2 specific inhibitor.^180^ However, further studies will be necessary to understand the real contribution of CK2 in the phosphorylation of these sites. In fact, none of them is surrounded by acidic residues, a general requirement for the CK2 phosphorylation (GGFG**S**_403_**S**_404_MDSK MDSK**S**_409_**S**_410_GWGM),^156^ and in vitro experiments suggest that CK1 could play the main role in the modification of these sites.^181,182^

In accordance with a protective role of CK2 in ALS, a disease-associated mutation has been recently identified in the *CCNF* gene, encoding the mutant S621G cyclin F protein. The mutation, found in a family with ALS and frontotemporal dementia (FTD), prevents the CK2-dependent phosphorylation. Cyclin F is part of the SKP1/cullin1/F‐box‐E3 ligase complex that promotes ubiquitination and degradation of many cellular proteins. Its phosphorylation by CK2 at S621 negatively controls the E3 ligase activity of the complex (Fig. 3e); consistently S621G mutation leads to the stimulation of the activity and the consequent aberrant increase of proteins ubiquitination, a hallmark of ALS and FTD.^183^

In general, the hypothesis of CK2 targeting in neurodegeneration is still premature. It is based on some observations, as in the case of ataxin-3 phosphorylation in SCA3 models,^175^ or SET phosphorylation^162^ and axonal transport inhibition^170^ in AD. Moreover, we found that CK2 phosphorylates HSJ1,^184^ a member of the DNAJ family of molecular chaperones, whose overexpression can reduce aggregation of neurodegeneration-associated proteins in vitro and in vivo. We showed that HSJ1 phosphorylation by CK2 is accompanied by a reduced ability of HSJ1 to bind ubiquitylated clients and to exert its chaperone activity. Thus, CK2 inhibitors would release the full neuroprotective potential of HSJ1.^184^

Very recently, in a cell-based model of tauopathies, recapitulating the abnormal deposition of phosphorylated tau protein, characteristic of several neurodegenerative disorders, the CK2 inhibitor TBB was identified as a potential drug candidate for its efficacy against tau hyperphosphorylation and oligomerization processes.^185^

However, there are also observations supporting roles for CK2 warning against its inhibition as a therapeutic strategy in neurodegenerative diseases.

### CK2 in infections

#### Viral infections

Several viruses exploit CK2 of the host cell for the phosphorylation of proteins, which, once phosphorylated, support the viral life cycle by multiple mechanisms (reviewed in^5^). Phosphorylation by CK2 was initially demonstrated for the human papillomavirus E7 protein,^186^ which, interestingly, is the target of the clinical grade drug CIGB-300, which, preventing its phosphorylation, displays promising anticancer properties (see above). Gradually, other substrates (as HIV-1 Rev protein,^187^ hepatitis C and B virus proteins^188,189^, and many others^5^) were added to the list of viral CK2 targets, which in 2003 already included ~40 proteins.^2^ New members are continuously added; many recently identified viral targets of CK2 are relevant for human diseases, as NSP1 of rotavirus (that causes severe diarrhea in young children),^190^ the nucleocapsid protein of the Hantaan virus (that causes serious disease syndromes in humans),^191^ the matrix M protein of human respiratory syncytial virus (that causes bronchiolitis and pneumonia in infants and the elderly),^192^ the leader L protein of the encephalomyocarditis virus,^193^ the Kaposi’s sarcoma-associated herpesvirus ORF57.^194^ Interestingly, this latter, when phosphorylated by CK2 in the proximity of a caspase-7 cleavage site, is no more cleaved, indicating that this classical antiapoptotic mechanism exerted by CK2 (see Fig. 2a) is valid also for viral proteins. It has to be considered, indeed, that viral infections have often pro-tumorigenesis functions.

CK2 inhibition appears as a promising strategy for antiviral therapy. Recently, Du and colleagues^195^ identified CK2 as a regulator of the TBK1/IFN regulatory factor 3 axis, which mediates the virus immune evasion to IFN response; consistently, they found that CK2 targeting increased IFN-α and IFN-β response, and elicited host defense mechanisms against virus infection. This finding suggests a therapeutic strategy that would not be restricted to a specific viral infection, but applicable to different DNA and RNA viruses.

In the context of the recent COVID-19 pandemic caused by Sars-CoV-2, it is worth it to mention that the inhibition of CK2 reduces the dysregulated production of inflammatory cytokines in response to SARS-CoV, another member of this virus family.^196^ The mechanism has been identified in the prevention of the CK2-dependent phosphorylation of the SARS-CoV receptor ACE2 (angiotensin-converting enzyme 2). SARS-CoV-2 exploits the same receptor, raising the interest in CK2 as an anti-COVID-19 target. Indeed, very recently, CK2 has been directly correlated to SARS-CoV-2 infection: it interacts with the viral protein N,^197^ and a number of CK2-dependent phospho-sites are found upregulated in infected cells, belonging to both viral and host cell proteins.^198^ Moreover, the clinical grade CK2 inhibitor CX-4945 has been tested for its antiviral efficacy in vitro and is considered as a potential anti-COVID-19 treatment.^199^

#### Bacterial infections

CK2 has been found important in increasing the invasiveness and motility of gastric cells infected by *Helicobacter pylori*,^132^ and therefore involved in determining the carcinogenesis process. Due to its regulatory role in NFκB-dependent inflammatory response, CK2 is implicated in polymicrobial infections.^200^ *Neisseria gonorrhoeae* (the etiological agent of gonorrhea),^201^ *Listeria monocytogenes* (which may cause infections of the nervous system)^202^, and *Candida albicans* (vaginal, oral, and skin candidiasis)^203^ are other bacteria whose infection implicates the host CK2.

#### Parasite infections

As far as parasites are concerned, the role of CK2 in cattle parasitosis by *Theileria* has been known for several years.^204^

It has been reported that CK2 is released, together with other protein kinases, during the life cycle of *Leishmania donovani*,^205^ and that this might play a role in parasite survival and adaptation to host environments. CK2 has been reported crucial also for *Leishmania braziliensis*, since its inhibition decreased virulent parasite growth,^206^ while *Leishmania tropica* (causing cutaneous leishmaniasis) exploits activation of CK2 thorough platelet-activating factor release.^207^

*Trypanosoma cruzi* is the causative agent of Chagas’ disease. It has been reported that human CK2, on the parasite cell surface, can phosphorylate proteins involved in cellular infection, which is consistently blocked by CK2 inhibitors.^208^

In *Schistosoma mansoni*, CK2 has been found to phosphorylate HMGB1 (high-mobility group box 1), a nuclear factor that can be secreted and acts as a cytokine.^209^ It has been proposed that targeting this phosphorylation might block HMGB1 secretion and therefore interfere with the pathogenesis of schistosomiasis.

*Toxoplasma gondii* might cause serious encephalitis in humans. Actin polymerization, which is crucial to parasite motility and host cell invasion, is controlled by CK2.^210^

Special interest has been dedicated to CK2 of the malaria parasite *Plasmodium falciparum*: it is crucial for the life cycle of the parasite, and its peculiarity compared to human CK2 offers opportunities to the development of specific inhibitors, thus making CK2 a potential target for antimalarial drugs.^211^ In turn, CK2 of the host erythrocyte has been also proposed as a target to prevent cytoadherence of *P. falciparum*-infected cells.^212^

### CK2 in ophthalmic diseases

Pathological angiogenesis in the retina is a major cause of visual impairment. CK2 has been found to mediate retinal vascularization and stem cell recruitment in a mouse model of oxygen-induced proliferative retinopathy; moreover, CK2 targeting reduced retinal angiogenesis and decreased normal and diabetic proliferation, migration, and viability of retinal endothelial cells.^213,214^ The angiogenic function of CK2 has been also found important for choroidal neovascularization in a mouse model of macular degeneration, since it mediates vascular endothelial growth factor production.^215^

Moreover, CK2 has been suggested as a therapeutic target after optic nerve injury, since its inhibition promotes retinal ganglion cell survival and axonal regeneration in rats.^216^

However, CK2 has also important functions in the eye: it is a component of the photoreceptor ciliary complex during dark adaptation of photoreceptor cells,^217^ and crucial proteins of both molecular motors and cytoskeletal components require phosphorylation by CK2 in developing retina.^218^ This should be considered while planning CK2 targeting for ophthalmic diseases.

### CK2 in cystic fibrosis (CF)

CF is an autosomal recessive inherited disorder that mainly affects Caucasian populations; until the 1980s, it was considered a pediatric disease but, nowadays, both the better knowledge of pathology and the improved therapeutic approach significantly enhanced patients’ quality of life, increasing the life expectancy to >40 years.^219^ CF is caused by mutations in *CFTR* (cystic fibrosis transmembrane conductance regulator) gene, which encodes the cAMP-regulated transmembrane channel CFTR, implicated in the transport of different ions (mainly chloride and HCO_3_^−^) and thus playing a crucial role in regulating the homeostasis of the lining fluids of the epithelia in which it is expressed. The 85% of the ~2000 alterations that have been characterized in the *CFTR* gene (which include frameshifts, insertions, deletions, and missense mutations) are disease-relevant, causing the complete lack of the channel or the synthesis of a defective protein with an impaired localization and/or activity. Among these alterations, the deletion of the phenylalanine residue 508 (Phe508del) is by far the most common, with a frequency of 70% in homozygosis and ~90% in heterozygosis with other *CFTR* mutations.^219,220^ The deletion of Phe508 residue causes CFTR misfolding and, in turn, prevents the channel from reaching the plasma membrane; in fact, over the 99% of the protein is trapped in the endoplasmic reticulum and prematurely undergoes proteasomal degradation. Worthy of note, the small fraction that escapes from ER shows altered gating activity, as well as a shorter half-life, compared to its w.t. counterpart.^220–222^

Recently, it has been suggested that a complex network of PTMs could be involved in CFTR fate, playing a pivotal role in determining its conformation, localization, turnover, and activity.^223,224^ In this scenario, it has been shown that also CK2 can directly interact and regulate the conductance of the w.t. CFTR, but not of the Phe508del mutant, and that CK2 inhibition effectively hampers the w.t. channel gating.^225^ However, although not effective in Phe508del-CFTR rescuing per se, CK2 inhibition mediated by epigallocatechin gallate was able to potentiate the effect of cysteamine, a proteostasis regulator that has been shown to promote CFTR maturation.^226^ Interestingly, an in vitro analysis highlighted a possible mutual mechanism of regulation between CFTR and CK2. It was shown that two residues on the NBD1 domain of the channel, namely, Ser422 and Ser670, were phosphorylated by CK2; intriguingly, small peptides from Phe508del-CFTR allosterically promote CK2 activity, implying that in CF, where a strong CFTR fragmentation is observed, the activity of the kinase could be upregulated.^227,228^

Most recently, we performed an in-depth analysis trying to solve several undisclosed issues concerning the CK2/CFTR relationship. By using both primary and immortalized bronchial epithelial cells, we did not report a direct correlation between CK2 expression/activity and CFTR rescue and, most importantly, we failed to find any significant difference in CK2 expression, activity and signaling by comparing primary cells from CF and healthy donors, suggesting that CK2 signaling is not altered in CF disease. Furthermore, we showed that the α′ catalytic subunit of CK2 is somehow involved in the halide channel rescue mediated by pharmacological chaperones belonging to class 1-correctors, which, through a direct interaction with CFTR, promote its folding and translocation to the plasma membrane.^229^ Our data were further supported by the work of Pankow et al.: by a detailed MS analysis, authors revealed that, when compared to its w.t. counterpart, Phe508del-CFTR undergoes different PTMs that prevent its maturation and translocation to the plasma membrane. Most importantly, a minimal PTM pattern has been identified in the NBD1-flanking region of CFTR, that has been suggested to act as a signature allowing the quality control of the endoplasmic reticulum, to discriminate a folded channel from one that is not; this “signature”, consisting in methylations and phosphorylations, is partly generated by CK2, thus further corroborating the hypothesis that CK2 activity should be preserved, rather than inhibited, for Phe508del-CFTR proper maturation.^224^ However, further investigation will be needed to substantiate whether the generation of the PTM code is functionally required for the CFTR maturation or if it is a secondary effect occurring in the biogenesis process of the protein.

Finally, it should be mentioned that CK2 has been related to the regulation of the Cl^−^/HCO_3_^−^ exchanger SLC4A2, an anion transporter expressed on the basolateral membrane of airway epithelial cells, deeply involved in regulating intracellular and extracellular pH, as well as chloride homeostasis. Numerous CK2 consensus motifs have been identified on SLC4A2 aminoacidic sequence; most importantly, the exposure of both Calu-3 cells (a human lung cancer cell line) and primary human nasal cells to the CK2 inhibitor CX-4945 led to the almost complete inhibition of channel activity.^230^ In the same way, it was shown that also the activity of TMEM16A, a Ca^2+^-activated chloride channel, is positively regulated by CK2; in fact, the TMEM16A conductance in CFBE airway epithelial cells is strongly hampered by both the pharmacological inhibition (mediated by TBB and CX-4945) and the transient downregulation of the α′ subunit of the kinase.^231^ These data further support the strong CK2 involvement in chloride and hydrogen carbonate homeostasis, that in the airway epithelia is fundamental for adequate solubilization and excretion of mucus, as well as for efficient antimicrobial action.

### CK2 in psychiatric disorders

A psychiatric disorder is a mental illness causing significant distress or impairment of personal functioning. Among the many substrates of CK2, several reports concern proteins implicated in mental illnesses, as described below.

#### Autism

It has been demonstrated that CK2 interacts with AUTS2, a component of the PRC1.5 complex.^232^ AUTS2, encoded by the autism susceptibility candidate 2 gene (*AUTS2*) is a nuclear protein expressed in the developing cerebral cortex and cerebellum regions often affected by neuropathological alterations in autism, and its mutations or disruption alter the transcriptional programs associated with normal brain development. In the PRC1.5 complex, AUTS2 interacts with PRC1 (Polycomb repressive complex 1), a major member of the Polycomb group (PcG) proteins, which maintain repressive forms of chromatin and appropriate patterns of gene repression through epigenetic mechanisms. PRC1, in particular, catalyzes the monoubiquitination of histone H2A at lysine 119. It has been demonstrated that CK2, in the PRC1.5 complex, directly interacts with AUTS2, modulating its interaction with other components of the complex (as P300); moreover CK2, by phosphorylating the RING1B member of the PRC1.5 complex, prevents the PRC1-mediated H2A monoubiquitination.^232^

A further link between CK2 and autism is represented by the phosphorylation of the fragile X mental retardation protein (FMRP). FMRP is an mRNA-binding protein, highly expressed in the brain and reproductive organs, that regulates the translation of mRNAs involved in neuroplasticity. The absence of FMRP in the neurons, due to altered expression of a single gene located on the X chromosome, causes the Fragile X syndrome, the leading monogenic cause for autism spectrum disorders. The role of FMRP in translation and plasticity is dependent on the phosphorylation of its Ser499. The kinase responsible for this phosphorylation is debated. However, a paper proposes CK2 as the main responsible kinase, which, by phosphorylating Ser499, would also allow the subsequent phosphorylation of other sites by different kinases.^233^

#### Attention deficit/hyperactivity disorder (ADHD)

ADHD is the most common neurodevelopmental disorder in children whose symptoms can persist in adulthood. It frequently accompanies Tourette syndrome (TS), a neurodevelopmental motor disorder. Slitrk1 is a transmembrane protein, highly expressed in the central nervous system, whose alterations have been identified in subjects with TS and also associated with ADHD. Slitrk1 is important for the formation of excitatory synapses between hippocampal neurons and for neuritogenesis, and its ablation leads to increased anxiety-like behavior. The correct function of Slitrk1 requires its interaction with 14-3-3 proteins. CK2 has been demonstrated crucial for the phosphorylation of Slitrk1 at the 14-3-3 proteins binding site and subsequent interaction, with functional consequences on the SLITRK1-induced neuritogenesis.^234^

#### Schizophrenia

The first finding of CK2 implication in schizophrenia dates back to 1991, when Aksenova and coworkers showed a reduction in soluble CK2, as well as an alteration of the phosphorylation pattern of some CK2 endogenous substrates in frontal brain cortex samples from schizophrenic and AD patients.^235^ Later, the analysis of prefrontal cortex samples from 15 schizophrenia patients confirmed a reduction of CK2α protein level, and in parallel highlighted a decreased phosphorylation of syntaxin 1, a CK2 substrate implicated in synaptic transmission.^236^ The reduced CK2-dependent phosphorylation of syntaxin 1 affects its ability to form SNARE complexes, altering the neurotransmitter release, thus contributing to the pathophysiology of schizophrenia.^236^ Interestingly, the paper reports also that antipsychotic drugs (APDs) seem to increase both CK2 and syntaxin 1 phosphorylation, suggesting that their therapeutic action may be related to this mechanism of action.

#### Major depressive disorder (MDD)

MDD, also referred to as clinical depression, is a common mental disorder that can affect many areas of life. The already mentioned study on schizophrenia also investigated CK2 and syntaxin 1 phosphorylation in 12 cases of MDD, without observing any reduction as found in schizophrenic patients.^236^ Indeed, a different study points to CK2 as a possible target in depressive disorder: it was found implicated in the regulation of the 5-HT_4_ receptor, a serotonin receptor emerging as an antidepressant therapeutic target.^237^ Rebholtz and her group showed that the 5-HT_4_ receptor is regulated by CK2, at transcriptional and posttranscriptional levels. Consequently, CK2α knockout mice overexpress the 5-HT_4_ receptor in the prefrontal cortex, and exhibit a robust antidepressed-like phenotype.^237^ Moreover, in vitro CK2 inhibition or knockdown enhance signaling and membrane localization of the 5-HT_4_ receptor. The mechanism is still unknown, but it is hypothesized that CK2 represses 5-HT_4_ receptor transcription by phosphorylating and regulating a yet to be identified transcription factor. In conclusion, CK2 activity in the prefrontal cortex is proposed as highly relevant in mood- and depression-related behaviors, and its targeting is suggested for the treatment of depression.

In summary, the role of CK2 in psychiatric disorders is multiple and complex, and it needs further investigation to assess to which extent and in which specific diseases it could be relevant for therapy.

### CK2 in Okur–Chung neurodevelopment syndrome (OCNDS)

For a long time, no pathogenic mutation of any of the CK2 subunit genes was known, until 2016, when Okur and coworkers analyzed 4102 intellectual disability/developmental delay cases by a whole-exome sequencing (WES) approach, and identified five patients from independent families with de novo missense and canonical splice site mutations in *CSNK2A1*, encoding the CK2α subunit.^238^ The patients shared overlapping neurodevelopmental disorders and dysmorphic features, a pathological condition that was hence defined as OCNDS (OMIM number 617062).

The missense variants involved residues located in regions important for CK2 activity and highly conserved across species. We performed a study for the kinetic analysis of four of these site mutations initially identified in OCNDS patients, namely Arg47Gln, Lys198Arg, Asp175Gly, and Tyr50Ser; interestingly, we found that they are associated with a general reduction of CK2 activity (Sarno et al., manuscript in preparation).

Several other de novo mutations in OCNDS patients were found later. A case was reported in 2017.^239^ In 2018 Chiu and colleagues reported on new eight OCNDS patients and summarized data on the six previously reported ones.^240^ Further 11 cases were described in the same year by Owen and colleagues,^241^ who identified congenital heart abnormalities in nearly 30% of the patients, and indicated this feature as a newly recognized *CSNK2A1* clinical association. They noticed that, while the majority of variants were identified in only one individual, the c.593A > G mutation causing the Lys198Arg variant was present in four unrelated individuals, and was therefore indicated as a hotspot for this syndrome. The Lys198Arg mutation was identified also in an 8-year-old Japanese boy showing a phenotype resembling Kleefstra syndrome (severe intellectual disability and synophrys), a syndrome that can be caused by haploinsufficiency of EHMT1 gene, encoding a histone methyltrasferase.^242^ The overlapping phenotype of the two syndromes could be partially related to a role of CK2 in controlling histone methylation, as hypothesized on the bases of our findings, showing that CK2 phosphorylates the demethylase LSD1,^243^ with potential regulatory functions.

A correlation between OCDNS and retinal dystrophy has been found in a 12-month-old male. The patient presented the mutation c.1061-1G > C located in the last coding exon not transcribing for CK2α, but important for mRNA maturation and stability.^244^

New insights on the effects of *CNK2A1* mutations come from a recent paper reporting of a novel mutation, Tyr50Cys, in a 5-year-old girl; while her clinical features were compatible with OCNDS, there were also duplication of the pituitary gland, absence of the olfactory bulbs, and multiple duplications of cervical vertebrae.^245^

The first case of vertical transmission from parent to child of a *CSNK2A1* variant was reported in a 6-year-10-month-old boy, who showed the Lys198Arg OCDNS mutation, but also a novel variant of TRPS1 gene, which encodes a zinc finger, GATA-type transcription factor that represses GATA-regulated genes and whose defect causes the tricho-rhino-phalangeal syndrome type I (TRPSI).^246^

Apart from CK2α variants, OCDNS has been found associated also to CK2β variants, providing evidence of a major role of this not-catalytic subunit on in vivo CK2 functionality. In 2019 a Japanese group identified four patients with neurodevelopmental disorders presenting de novo variants in *CSNK2A1*, but also in *CSNK2B* genes.^247^ All patients showed intellectual disabilities and developmental delays. Consistently, *CSNK2B* splice site mutations and truncating mutations were reported in patients with intellectual disability by other groups,^248,249^ who also suggested a possible correlation of CK2β variants with epilepsy (see below, POBINDS).

Up to now, ~60 patients worldwide have been diagnosed with OCDNS, but it is expected that more cases will be disclosed by the increasing utilization of WES approach.

A nonprofit organization, the *CSNK2A1* foundation, has been established in 2016, with the scope of finding a cure for OCNDS and supporting patients (https://www.csnk2a1foundation.org/).

### CK2 in Poirier–Bienvenu neurodevelopmental syndrome (POBINDS)

Mutations of the CK2β gene (*CSNK2B*) have been recently found, associated with a pathological condition, called POBINDS (OMIM number 618732). This is a newly defined neurologic disorder, mainly characterized by early-onset seizures and/or intellectual disability/development delay, recently described as an autosomal dominant inherited disease, caused by heterozygous mutation in the *CSNK2B* gene.^247–250^ In the last 3 years, 14 *CSNK2B* de novo variants associated with POBINDS were found with trio WES and they were classified as deleterious (CADD scores) and pathogenic (ACMG guidelines).^247,250^ The *CSNK2B* mutations associated with POBINDS were localized in different part of the gene, some of them generating a truncated form of CK2β, others inducing single amino acid changes in functional protein domains. A *CSNK2B* haploinsufficiency emerged in some patients, but the functional role of the *CSNK2B* variants remains still unknown.

Of course, pharmacological targeting of CK2 with inhibitors cannot be considered a strategy for OCNDS and POBINDS patients, who very probably suffer from a low phosphorylation degree of CK2 substrates (or of some of them), due to defective CK2 activity (Sarno et al., manuscript in preparation). However, we decided to consider these pathologies in this review, since we think that the readers need to be aware of them, also in order to promote interest and encourage investigation on this relatively new field.

### CK2 in diabetes and obesity

Diabetes mellitus (DM), usually known as diabetes, is a metabolic disease characterized by chronic hyperglycemia, whose worldwide prevalence has been rapidly rising in the past decades.^251^ The main subtypes of DM are type 1 diabetes mellitus (T1DM), which is associated to a defective insulin secretion due to an autoimmune pancreatic β-cell destruction, and type 2 diabetes mellitus (T2DM), associated to progressive loss of β-cells functionality and insulin resistance,^252^ frequently accompanied by obesity.^252^

To date, a significant number of studies have reported a potential role of CK2 in the modulation of diverse pathways associated with the development of DM, proposing this kinase as a possible target for DM therapy.^253^ However, quite distinct and sometimes controversial findings have been reported, possibly due to the different tissues where the CK2 functions were evaluated. For example, an alteration of CK2 kinetic properties was initially associated with DM in very early studies on the liver and skeletal muscles of streptozotocin (STZ)-diabetic rats, a model of T1DM.^254,255^ In the liver, the analysis of cytosolic CK2 activity revealed a decrease of its *K*_m_ for various substrates in the diabetic rats compared to their control, with the possible consequence of a higher phosphorylation level of those substrates.^254^ Also, the same study demonstrated that the administration of insulin to the diabetic rats was able to revert the alteration of the CK2 *K*_m_. On the opposite, in the skeletal muscle of STZ-diabetic rats, the specific activity of CK2 was found decreased in comparison to control rats.^255^ A more recent study, based on the MS proteomic profile of pancreatic islets from nonobese diabetic (NOD) mice, evidenced a marked reduction of CK2 catalytic subunits compared to healthy samples.^256^ On the contrary, CK2α was found highly expressed and hyper-activated in ventricular cardiomyocytes of STZ-diabetic rats and in high-glucose-treated H9c2 cells in parallel with the augmented expression of the Zn^2+^ transporters ZIP7, which is mainly localized in the sarco(endo)plasmic reticulum (S(E)R). The CK2α upregulation was described as crucial for the phosphorylation of ZIP7, which in turn caused a rise of cytosolic free Zn^2+^ and a decrease of S(E)R free Zn^2+^ in hyperglycemic conditions.^257^ Consistently, the CK2α downregulation, by reducing the ZIP7 phosphorylation, was found to revert the Zn^2+^ redistribution.^257^ Notably, in the heart of T1DM rats, CK2α protein level was found increased in the S(E)R and decreased in the nuclei compared to normal rats, suggesting that the diabetic cardiomyopathy condition could induce a redistribution of intracellular CK2α from the nuclei to the S(E)R.^258^

In a study on skin fibroblast from T1DM patients with (T1DM+) or without (T1DM−) nephropathy and healthy subjects, we analyzed the possible correlation of CK2 with diabetic nephropathy. We did not observe differences in CK2 protein level among the groups; interestingly, however, T1DM+ cells showed a lower CK2 activity but were more sensitive to CK2 inhibitors, compared to the other groups, in terms of cell death induction.^259^ The relevance of CK2 in the in vivo pathological process of diabetic nephropathy was further demonstrated by Huang et al.,^260^ who found an upregulation of CK2 in the kidney of two different mouse models of T2DM, and showed that CK2 reduction/inhibition in STZ-diabetic rats and mice ameliorated renal fibrosis. The identified mechanism relies on the regulation of the expression in glomeruli of diabetic renal fibrotic factors, such as fibronectin and intercellular adhesion molecule-1, under the control of CK2 through NFκB.^260^ Furthermore, the overexpression of sphingosine kinase 1 was suggested as the mediator of the aberrant CK2 action in the diabetic renal inflammatory fibrosis via NFκB pathway.^261^

The involvement of CK2 in the development of diabetes-associated retinopathy has been also hypothesized, as discussed above^214^ and reviewed by Ampofo and colleagues.^253^

In T2DM, alteration of CK2 activity and expression were also associated with impaired β-cell function and insulin resistance. In fact, the analysis of human microarray data of pancreatic β-cell enriched samples from T2DM patients and healthy donors revealed a significant rise of ∼1.2-fold in CK2α gene (*CSNK2A1*) expression in T2DM subjects, compared to nondiabetic individuals.^262^ In addition, the comparison of liver tissues between obese T2DM mice and control mice aged of 16 and 32 weeks highlighted that *CSNK2A1* expression and CK2α protein level are strikingly increased in diabetic animals. Importantly, this study analyzed also human patients; although the small number of analyzed individuals did not reach the statistical significance, the CK2α amount in the serum of diabetic patients was found higher than that in the healthy donors, suggesting that CK2 is upregulated in obese T2DM subjects.^263^ The overexpression of CK2α detected in obese T2DM mice^263^, and the downregulation of CK2α and CK2α′ observed in NOD mice^256^ suggest a possible involvement of CK2 in the development of the obesity phenotype. Other studies supported the evidence that the obesity condition per se, independently of diabetes, could be associated with alteration of CK2 signaling. Indeed, the analysis of liver tissue of insulin‐resistant obese rats (fa/fa model) and lean rats highlighted a change in CK2 distribution between the cytosol and the membranous fractions, with decreases in the cytosol and increases in the membranes of fa/fa rats compared to control rats.^264^ Similarly, we found that CK2 activity and protein level were upregulated in the adipose tissue (AT) of obese and obese T2DM mice compared to their relative controls.^265^ Moreover, the analysis of AT from different obese human subjects highlighted that CK2 was higher in samples from patients than from normal-weight controls, independently of their insulin resistance severity or the presence of T2DM. Of note, CK2 alterations reverted to physiological level in AT of obese patients who underwent a significant weight loss. Altogether, these findings support the hypothesis that aberrant CK2 signaling could be strictly associated with the pathophysiology of AT related to obesity rather than T2DM.^265^

Shinoda et al., by analyzing the CK2 activity in the ATs of mice under a high-fat diet or a regular diet, demonstrated that CK2 activity increased in response to obesogenic diet.^266^ In addition, they found that the genetic and pharmacological targeting of CK2 ameliorated the diet-induced obesity and insulin resistance in mice, and promoted the UCP1-dependent thermogenesis in vivo, by reducing the CK2-mediated phosphorylation of class I histone deacetylases (HDACs).^266^

CK2 was shown to be essential also for the adipogenic differentiation^267,268^ and, in particular, for the mitotic clonal expansion, one of the initial events of adipogenesis.^269^ In more details, it was demonstrated that the deacetylase sirtuin 6 (SIRT6) is able to promote adipogenesis by reducing the expression of the KIF5C (kinesin family member 5C), a negative regulator of the adipogenic process.^269^ The downregulation of KIF5C, a known binding partner of CK2α′,^270^ promoted the nuclear translocation of CK2, favoring the mitotic clonal expansion.^269^ Moreover, we demonstrated that CK2 positively modulates the adipocyte insulin-stimulated glucose uptake.^265^ It could be therefore speculated that the upregulation of CK2 in the AT of obese patients is the result of a selective pressure, awarding efforts to increase the storage capacity of white adipocytes, favoring their glucose uptake, and leading to sustain adipogenesis essential for the AT expansion. Recently, a possible role of CK2 was suggested in the development of multiple symmetric lipomatosis (MSL), a rare disorder characterized by the growth of nonencapsulated masses of subcutaneous adipose tissue (SAT). In particular, the analysis of the lipoma samples in comparison to healthy SAT specimens from the same MSL patient evidenced a CK2 hyperactivation, in parallel with an upregulation of Akt and ERK1/2 signaling, in the lipomatous tissue.^271^

The possibility that insulin regulates CK2 was largely debated^272–278^; a reevaluation in 1994 excluded this hypothesis.^279^ However, on the other side, different studies disclosed a possible CK2 involvement in the regulation of insulin expression and secretion. In pancreatic β-cells, CK2 was demonstrated to suppress insulin expression by phosphorylating the transcription factor PDX-1^280^ and its upstream regulators USF1 (upstream stimulatory factor 1)^281^ and MST1 (mammalian sterile 20-like kinase 1); consistently, CK2 inhibition was found to induce insulin expression.^282^ Furthermore, CK2 phosphorylates the β-cell M3Rs (G-protein-coupled muscarinic M3 receptors), modulating their activity in vitro and in vivo, and CK2 blockade, by reducing this phosphorylation, greatly promotes the M3R-stimulated insulin secretion in pancreatic β-cells.^283^ Moreover, the inhibition of CK2 was demonstrated to protect mouse and human islets from glucolipotoxicity, a process associated with T2DM that causes β-cell damage and dysfunction due to a chronic exposure to high glucose and lipids. On one hand, CK2 inhibition was suggested to revert the reduced acetylcholine-stimulated insulin secretion induced by glucolipotoxicity and correlated to the phosphorylation of M3Rs. On the other hand, CK2 downregulation seemed to counteract the β-granule phosphoprotein kinesin heavy chain phosphorylation and stimulate the insulin secretion by promoting β-granule transport along microtubules in pancreatic β-cells.^284^

In conclusion, there is strong evidence supporting roles for CK2 in DM, in its complications such as nephropathy and retinopathy, and in obesity, but several aspects need further investigation before planning therapeutic application of CK2 inhibitors for these diseases.

### CK2 in inflammatory diseases

The interest in CK2 as a possible target in inflammation is quite recent.^285^ It is now confirmed that CK2 inhibition suppresses the secretion of IL-6, and is therefore a potential therapy in diseases where IL-6 is instrumental, as rheumatoid arthritis.^286^ CK2 activity is higher in animal models of chronic colitis, where its nuclear translocation has been observed.^287^ In chronic intestinal inflammation, CK2-dependent activation of NFκB signaling is considered crucial.^288^ The regulation of NFκB is also instrumental for CK2 implication in renal inflammatory diseases, as diabetic fibrosis, which is ameliorated in diabetic animals by treatments with CK2 inhibitors.^260^ Renal injury due to glomerulonephritis has been also correlated to CK2, which is considered a potential target for this progressive inflammation, a primary cause of chronic renal failure.^289^

More recently, the mechanism by which CK2 controls immune response and its implication in autoimmune disorders has been detailed.^290^ In particular, CK2 promotes CD4^+^ T-cell proliferation and Th1 and Th17 responses, and this has been found crucial for the CK2 contribution to the pathogenesis of Crohn’s disease, suggesting CK2 inhibition as a novel therapeutic treatment for this pathology.^291^ Moreover, CK2 is necessary for the functions of claudin-2, a tight junction protein upregulated in chronic immune-mediated colitis, and in fact CK2 inhibition attenuates the diseases progression, but not in claudin-2-knockout animals.^292^

### Cardiovascular diseases

It was initially found that CK2 activity increased in ischemic preconditioning; however, the activation appeared to be an epiphenomenon and did not reduce the infarction from myocardial ischemia.^293^ Later, it has been demonstrated that CK2 is associated with the pathogenesis of cardiac ischemia–reperfusion injury and the dysregulated mitochondrial homeostasis through the suppression of FUNDC1-related mitophagy.^294^

CK2 inhibition prevents the accumulation of vascular smooth muscle cells within the neointimal compartment, a cause of accelerated atherosclerosis.^295^

A seminal paper was published by Hauck and colleagues, showing that p27 (KIP1), a well-known cell cycle regulator, requires CK2 to mediate pathologic growth of cardiomyocytes, with potential implications for the development of new approaches to treat cardiac hypertrophy.^296^ Moreover, it is worth it to mention that p27 protects cardiomyocytes by activating autophagy and inhibiting apoptosis,^297^ suggesting that CK2, through p27, could be relevant also for the autophagy-dependent growth of cardiomyocyte cells. Interestingly, the screening for proteins interacting with p27 in cardiomyocytes specifically identified the α′ catalytic subunit of CK2, and in fact α′ silencing abolished p27 phosphorylation, suggesting an isoform-specific function of CK2 in this pathology (although further studies will be necessary to exclude a similar contribution of α). It has been also reported that the CK2 contribution to the cardiac hypertrophy is due to the phosphorylation of HDAC2.^298^

However, there are also discrepant findings: CK2 has been defined as an anti-hypertrophic pathway, and found downregulated in cardiac hypertrophy.^299^ Moreover, it is worth noting that beneficial effects of CK2 have been reported in the cardiovascular system: CK2 is necessary for the physiologic hypertrophy of heart,^300^ its decreased interaction with the potassium channel SK2 is associated with heart failure,^301^ and its reduced expression contributes to neuronal apoptosis in cerebral ischemia–reperfusion injury.^302^

## Conclusions

**Fig. 4:**
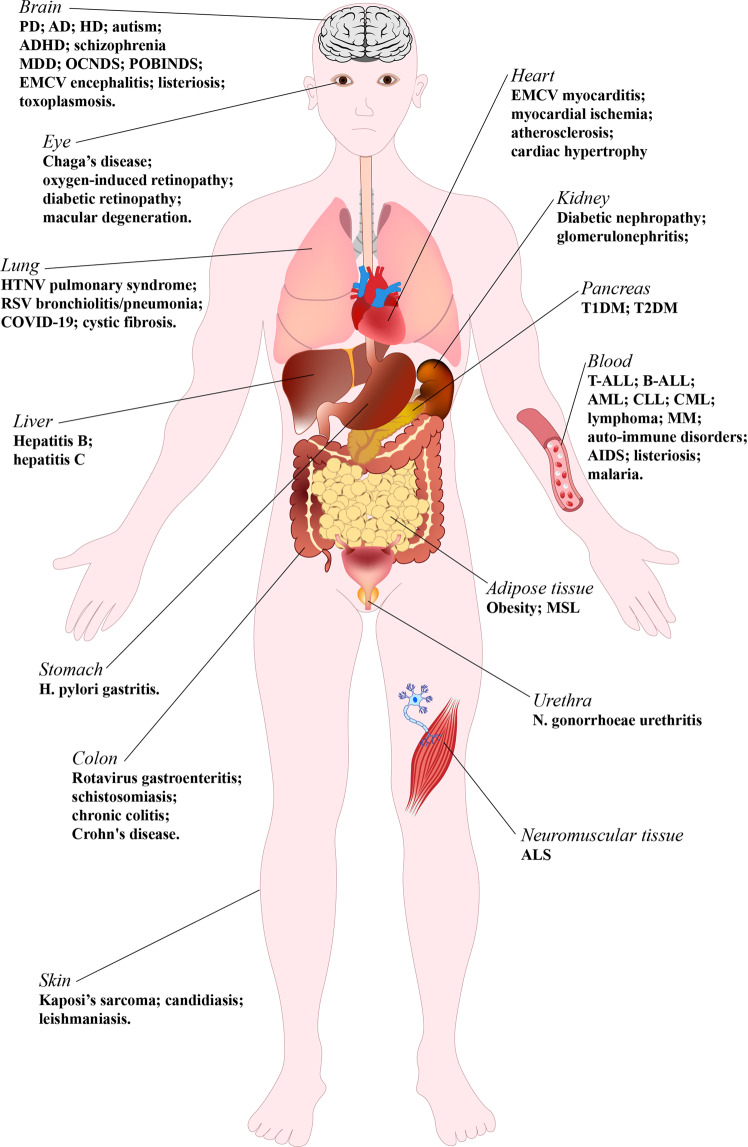
CK2-related human pathologies. Human organs/tissues are schematically represented as the main sites of diseases, where CK2 has been found implicated. Solid tumors are not included in this scheme. The following abbreviations are used: PD Parkinson’s disease, AD Alzheimer’s disease, HD Huntington’s disease, ADHD attention deficit/hyperactivity disorder, MDD major depressive disorder, OCNDS Okur–Chung neurodevelopment syndrome, HTNV Hantaan virus, RSV respiratory syncytial virus, COVID-19 coronavirus Sars-CoV-2 disease, EMCV encephalomyocarditis virus, T1DM type 1 diabetes mellitus, T2DM type 2 diabetes mellitus, T-ALL T-cell acute lymphoblastic leukemia, B-ALL B-cell acute lymphoblastic leukemia, AML acute myeloid leukemia, CLL chronic lymphocytic leukemia, CML chronic myelogenous leukemia; MM multiple myeloma, MSL multiple symmetric lipomatosis, ALS amyotrophic lateral sclerosis

The scenery depicted in this review highlights CK2 as a molecule of deep interest in many fields of human medicine. It is strongly implicated in the pathogenesis of several diseases, as illustrated in Fig. 4, where several CK2-related human pathologies are indicated on the organs/tissues of their main molecular localization and/or clinical manifestation. A detailed knowledge of CK2 functions in human diseases is fundamental either to plan its targeting as possible therapy or, in any case, to better understand the molecular mechanisms underlying the pathogenic processes and to plan the correct interventions for a better tailored therapy. For some diseases, CK2 can be definitely recognized as a drug target. In particular, this is the case of cancer: promising anticancer effects have been obtained with CK2 inhibitors in countless studies, in cellular and animal models (see Table 2), and clinical trials are ongoing with the CK2 inhibitors CX-4945 (https://clinicaltrials.gov/ct2/results?cond=&term=CX-4945&cntry=&state=&city=&dist=) and CIGB-300 (https://clinicaltrials.gov/ct2/results?cond=&term=cigb-300&cntry=&state=&city=&dist=) for different cancers. However, little is known on the bioavailability and pharmacokinetics of CK2 inhibitors; only preliminary studies have been published on CX-4945, although the in vivo data are encouraging.^303,304^ Moreover, it has been reported that CX-4945 can cross the blood brain barrier,^98,99^ but this issue should be better defined under physiological and pathological conditions. On the effects and mechanism of action of CX-4945, the induction of methuosis has been reported in colorectal cancer cells, associated with reduced tumorigenicity,^31^ but the CX-4945-induced methuosis has been described as independent of CK2 in cholangiocarcinoma cells.^305^

Concerning CK2 targeting in cancer, it is worth mentioning that, besides monotherapy with the kinase inhibitor, there is great expectation in combined therapies, which simultaneously target an onco-pathways (specifically abnormal in a certain cancer) and CK2, which more generally potentiates tumorigenic signals. Several studies have already been published, showing that CK2 inhibitors synergize with different antitumor drugs (reviewed in refs. ^37,42^). Interestingly, the strategy has been found successful also in case of drug resistance phenotype (reviewed in^42^). Presently, a combined therapy based on CX-4945 and Ku 60019 (an inhibitor of ATM kinase^306^) is under evaluation on organoid cultures of human renal tumors (ClinicalTrials.gov Identifier: NCT03571438). Future clinical applications of anticancer combined therapies exploiting CK2 inhibition are expected.

For other pathologies, like cardiovascular diseases, the actual feasibility of therapies based on CK2 targeting is less clear-cut, often due to controversial reports (see the specific paragraph for references). Some reports suggest a beneficial effect of CK2 inhibition, but conclusions are not unambiguous, yet, and evidence of CK2 physiological and essential functions should be also considered. Similarly, the hypothesis of CK2 targeting for AD, and in general for neurodegeneration (see the specific paragraph for references), is still premature (see Fig. 3). For CF, if initially CK2 inhibition had been suggested as a potential therapeutic intervention to potentiate the effect of proteostasis regulators for Phe508del-CFTR rescue,^226^ recent evidence suggests that CK2 activity is required for proper CFTR mutant maturation,^224,229^ and for the preservation of other ion channels that may compensate for CFTR dysfunctions.^231^

In the eye, CK2 inhibition prevents pathological retina angiogenesis,^213,214^ but, on the other side, several CK2-dependent phosphorylations are necessary for the correct eye development.^216,217^ Therefore, it has to be determined whether CK2 inhibition is appropriate to prevent the high proliferating rate required for abnormal angiogenesis, without affecting normal retinal functions.

In several psychiatric disorders, the implication of CK2 is undoubted, however, its activity might be either instrumental to the pathology (as for depression^237^), or instead necessary for allowing the correct physiological functions and preventing the disease (autism,^232^ ADHD,^234^ and schizophrenia^235,236^) and should be preserved or even increased to prevent the mental disease. Interestingly, APDs have been reported to increase CK2 activity and phosphorylation of syntaxin 1.^236^ This finding needs further investigation, since, should it be confirmed, it would indicate a therapeutic strategy to be evaluated also in other pathological conditions characterized by a too low CK2 activity.

The recently defined OCNDS and POBINDS are pathologies associated with loss-of-function mutations of the CK2 genes (see the specific paragraphs for references), and are among the conditions for which CK2 targeting should be aimed at increasing, instead of inhibiting, its activity. While a battery of CK2 inhibitors (Table 1) are already in the hands of scientists and even of clinicians (Table 2), no CK2 activator has been developed so far. Future investigations will disclose whether different approaches for CK2 targeting will be possible, not excluding genetic intervention.
